# Myokine Secretion Dynamics and Their Role in Critically Ill Patients: A Scoping Review

**DOI:** 10.3390/jcm14092892

**Published:** 2025-04-23

**Authors:** Yorschua Jalil, L. Felipe Damiani, Patricio García-Valdés, Roque Basoalto, Julen Gallastegui, Ruvistay Gutierrez-Arias

**Affiliations:** 1 Escuela de Ciencias de la Salud, Departamento de Kinesiología, Facultad de Medicina, Pontificia Universidad Católica de Chile, Santiago 1270709, Chile; yfjalil@uc.cl (Y.J.); lfdamiani@uc.cl (L.F.D.); pgarcs@uc.cl (P.G.-V.); julen.gallastegui@uc.cl (J.G.); 2 CardioREspirAtory Research Laboratory, Departamento Ciencias de la Salud, Pontificia Universidad Católica de Chile, Santiago 7820436, Chile; roque.basoalto@gmail.com; 3 Department of Intensive Care Medicine, Faculty of Medicine, Pontificia Universidad Católica de Chile, Santiago 1270709, Chile; 4 Departamento de Apoyo en Rehabilitación Cardiopulmonar Integral, Instituto Nacional del Tórax, Santiago 8320000, Chile; 5 INTRehab Research Group, Instituto Nacional del Tórax, Santiago 8320000, Chile; 6 Faculty of Rehabilitation Sciences, Exercise and Rehabilitation Sciences Institute, Universidad Andres Bello, Santiago 7591538, Chile

**Keywords:** critical care, critical illness, myokine, muscle contraction, rehabilitation

## Abstract

**Background/Objectives:** Myokines can modulate organ function and metabolism, offering a protective profile against ICU complications beyond preventing local muscle wasting. This scoping review aims to explore and summarize the evidence regarding the secretion of myokines and their potential local or systemic effects in critically ill patients. **Methods:** A scoping review following Joana Briggs Institute recommendations was conducted. A systematic search of MEDLINE (Ovid), Embase (Ovid), CENTRAL, CINAHL (EBSCOhost), WoS, and Scopus was conducted from inception to February 2023. We included primary studies evaluating myokine secretion/concentration in critically ill adults undergoing physical rehabilitation interventions. Two independent reviewers performed study selection and data extraction. **Results:** Seventeen studies published between 2012 and 2023 were included. Most were randomized clinical trials (47%). Physical rehabilitation interventions included electrical muscle stimulation, as well as passive and active mobilization, delivered alone or combined, in single or daily sessions lasting 20–60 min. Twelve studies (70%) evaluated interleukin-6, while interleukin-10, tumour necrosis factor-α, Interleukin-8, and myostatin were also commonly studied. Thirteen studies (76%) reported changes in myokine secretion or gene expression, although no clear concentration change pattern emerged. Myokines involved in muscle protein synthesis and breakdown may protect against muscle waste and weakness. **Conclusions:** The study of myokine dynamics in critically ill patients highlights the systemic impact of physical rehabilitation. This emerging field has grown in interest over the past decade, offering significant research potential. However, challenges such as study design, small sample sizes, and variability in physical therapy protocols hinder a comprehensive understanding of myokine responses.

## 1. Introduction

Critically ill patients hospitalised in the intensive care unit (ICU) are characterised by accelerated skeletal muscle wasting, which is partially attributed to sepsis, multiple organ failure, and immobilisation [1,2,3,4,5]. Since skeletal muscle is a highly organised tissue that participates in several body functions, such muscle insult gravitates at several levels, altering structural support, locomotion, breathing, metabolism, and energy supply to the body [6].

Muscle weakness affecting both the respiratory and peripheral muscles is thought to be the key mediator of disability after critical illness [1]. The development of ICU-acquired weakness (ICU-AW), a neuromuscular disorder characterised by profound peripheral muscle weakness and loss of physical functions, even after discharge, has been associated with delayed weaning from mechanical ventilation (MV), prolonged ICU and hospital stay, and increased mortality [2,3,4,5,7,8]. Respiratory muscle wasting occurs early (18 to 69 h) in up to 60% of patients with MV, rapidly leading to diaphragmatic weakness and dysfunction, and has also been shown to be an independent risk factor for prolonged weaning from MV and higher mortality [9,10,11]

The negative consequences of ICU-AW can be partially avoided through early and active physical mobilisation. However, its application at the early stages is challenging and requires a highly trained, multidisciplinary team. Passive limb mobilisation, mild degrees of muscle activation, and even external muscle contraction induced through neuromuscular electrical stimulation (NMES) represent alternatives to traditional exercise, as they are able to prevent local muscle wasting and potentially cause a systemic effect through a diverse range of cytokines and chemokines secreted by myocytes during muscle stimulation (i.e., myokines) [12,13,14,15,16].

It is thought that myokines can promote a protective profile for the development of several ICU complications that is not only beneficial for the local prevention of muscle wasting but that also modulates the function and metabolism of distant organs [17,18,19,20,21].

Although the potential role of myokines in critically ill patients is an emerging concept, the isolated secretion of some myokines has been previously reported in a general and indirect manner for this population. However, no research has focused on their secretion dynamics and the role that they could play in clinical practice as a biomarker in critical settings [22,23,24]. Understanding patterns in myokine secretion could contribute to the design of ‘better’ rehabilitation programs to improve physical function in critically ill patients.

This scoping review aims to explore and summarize the evidence regarding myokines’ secretion dynamics and their potential local or systemic effects in critically ill patients, especially in the early stages upon admission to the ICU. It is necessary to answer the following questions: (1) Which myokines are released during physical rehabilitation of critically ill patients? (2) Which physical rehabilitation modality is most beneficial for myokine release? (3) Can myokines exert local or systemic clinical effects in critically ill patients?

## 2. Methods

A scoping review was conducted following the updated recommendations of the Joanna Briggs Institute (JBI) [25]. The protocol for this review was registered on the International Platform of Registered Systematic Review and Meta-analysis Protocols (INPLASY) under number INPLASY202190048. The results are reported following the Extension for Scoping Reviews of the Preferred Reporting Items for Systematic Reviews and Meta-analyses statement (PRISMA-ScR) [26].

### 2.1. Data Sources and Searches

A systematic search was conducted until February 2023 in MEDLINE through the Ovid platform, Embase through the Ovid platform, the Cumulative Index of Nursing and Allied Literature Complete (CINAHL Complete) via the EBSCOhost platform, Web of Science (WoS), and Scopus. The strategy considered a sensitive approach, and controlled (MeSH, Emtree, and CINAHL Subject Heading) and natural language was used. It included terms related to myokines, population (critically ill patients), and physical interventions that produce muscle activation. Table 1 shows the search strategy used in the MEDLINE database (Ovid). Refer to the Supplementary Material for specific terms used in each database (See Supplementary Table S1).

In addition, we searched the references of the included studies using the Citationchaser backward search [27] and identified reports that cited the studies included in this scoping review using the Citationchaser forward search [27].

### 2.2. Eligibility Criteria

Eligibility criteria for study selection were divided into participants or populations included in the studies, the concept or phenomenon involved, and the context in which the studies were conducted (PCC framework) [25]. In addition, study designs were considered for inclusion in this review as follows:

Participants: We included studies that enrolled adult critically ill patients (18 years or older) with or at risk of acquiring any neuromuscular disorder related to loss of muscle function, independent of the ventilatory support strategy and the type of ICU in which they were hospitalised (intensive, intermediate, medical, surgical, cardiac, or mixed, among others).

Concept: Studies assessing the secretion/concentration of myokines or similar peptides (exerkines, cytokines, or interleukins) attributable to muscle stimulation achieved by any intervention (physical activity, exercise, and NMES, among others) were included. These interventions were defined as any activity in which myocytes can be stimulated because of muscle activation, regardless of the frequency, intensity, time of application, and muscle being stimulated.

Context: In critical care settings, the potential effects of myokines were assessed locally or systemically. Results could be reported regarding muscle structure or function, such as skeletal muscle mass and/or strength, the degree of muscle wasting, or myopathies.

Study designs: Only primary studies (randomised controlled trials, cohort studies, case–control, cross-sectional, and case reports) were included. Available full texts or conference abstracts were included. The language and publication date of the studies did not limit their inclusion.

### 2.3. Study Selection

After searching for studies, two independent reviewers screened titles and abstracts, discarding studies irrelevant to this review. First, we piloted the completeness and clarity of the eligibility criteria of the studies for the first 100 records, making minimal adjustments after this test. Subsequently, the same reviewers assessed the full text of potential studies for inclusion, utilising the Rayyan^®^ application (Copyright© 2025 Rayyan) [28]. Disagreements were resolved through consensus in the first instance, and in cases where consensus could not be reached, a third reviewer made the final decision regarding inclusion of studies.

### 2.4. Data Extraction

Two reviewers independently extracted information from the included studies. An extraction form sheet specifically designed to meet the objectives of this review was used and developed in a Microsoft Excel^®^ spreadsheet (Microsoft Excel^®^ (Version 2501)). Information was extracted about study identification, the included population, applied interventions, myokine secretion and its assessment method, and outcome data relating to any effect of myokine secretion (local or systemic). Disagreements were resolved by consensus in the first instance. A third reviewer confirmed the data in cases where this was not possible.

### 2.5. Data Synthesis

The search results and study selection process were documented using a PRISMA flow chart [26]. Additionally, a table was prepared explaining the reasons for excluding evaluated full-text studies. Results are presented narratively, incorporating tables and figures to synthesise information.

## 3. Results

### 3.1. Study Selection

**Figure 1:**
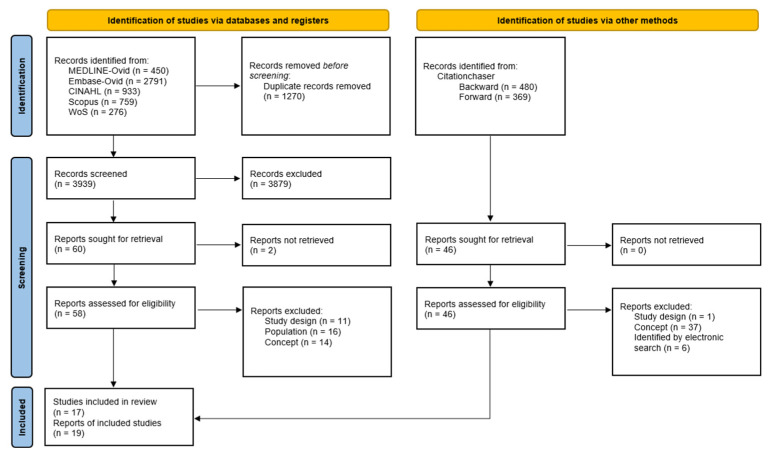
PRISMA flow diagram.

The search identified 3939 unique records, of which 3879 were considered irrelevant. Of the 60 studies assessed in full text, 16 (18 reports) met the eligibility criteria. In addition, the backward–forward search identified one study and one additional report (Figure 1). Therefore, this scoping review included 17 primary studies published in 19 reports [29,30,31,32,33,34,35,36,37,38,39,40,41,42,43,44,45].

The primary reasons for exclusion included the type of study (especially literature reviews), the inclusion of a non-critically ill population, inadequate assessment of myokine concentration in relation to muscle activation, and the absence of myokine concentration as outcome (See Supplementary Table S2).

### 3.2. Study Characterisation

The 17 included studies were published between 2007 and 2023, with almost half (10 studies; 41%) published recently, from 2020 to 2023. The most frequent years of the reports were 2015 and 2020, with two articles each. Predominantly, the studies come from the United States (6 articles; 35%) [32,33,34,35,41,44], Brazil (4 articles; 24%) [36,39,40,42], and Germany (2 articles; 12%) [30,31]. The most common study design was a randomised controlled trial (RCT) (8 articles; 47%), followed by prospective cohort studies (6 articles; 35%). Two studies were considered retrospective [29,30], as they analysed data obtained from previous clinical trials (prospective and randomised). Additionally, one study was quasi-experimental [44]. See Table 2 for further details.

### 3.3. Population

Participants included in the studies varied in sample size and characteristics. Sample sizes were selected mainly by convenience, ranging from 11 [42] to 100 patients [41], with nine studies being under the 50-patient mark [33,34,38,39,40,42,43,44,45]. Some reports included patients within 48–72 h from intubation and MV initiation [31,32,34,44,45], while other authors focused on the chronic critically ill scenario (patients with 10 days on MV [33]).

The physio-pathological context also varied, considering COPD patients [45], patients admitted to a specialist cardiothoracic ICU before elective high-risk cardiac surgery [43], acute respiratory failure patients [41], septic patients [37], and traumatic brain injury patients [36]. In particular, the results presented by Grunow et al. [30] and Vanhorebeek et al. [29] were pooled from data reported in other clinical studies: two prospective and two randomised clinical trials, respectively. These studies included 12 to 162 ICU patients. The latter study, corresponding to an RCT comparing two nutritional strategies related to the timing of initiating supplemental parenteral nutrition, was the largest one. This study provided standard physiotherapy to all patients, without further comparison regarding the effect of exercise [29].

### 3.4. Physical Intervention

Nine studies included electrical muscle stimulation as an intervention. Seven of them incorporated NMES [29,30,31,36,37,43,45] or functional electrical stimulation (FES) [38,40]. Some authors integrated NMES as part of a multimodal intervention in the rehabilitation context. Kayambu et al. [37] and Silva et al. [36] included passive and active motions, with early mobilisation, along with NMES.

Three studies exclusively employed passive exercise as an intervention, primarily through an ergometric cycle [39,42,44]. This approach was also included in seven other studies [31,32,33,36,37,40,41], as part of a “rehabilitation session”, frequently including active movement in an early progressive mobility protocol. This “multimodal rehabilitation” approach was the most provided intervention among all studies (71%).

Exercise prescription varied in terms of intervention modality and program. Some studies provided a single-session exercise [39,40,42,44], mainly focusing on passive mobility. Other studies provided exercise sessions over weeks, even until ICU discharge [32,37], once or twice a day according to study protocol, with the most common setting being two sessions per day [33,37,38,41,43] compared to only once [31,34,35,36].

Body activation, including upper- and lower-extremity muscles, in the context of multimodal rehabilitation, was the most frequently targeted [32,33,34,35,36,37,41]. Specific lower-extremity interventions were also common, such as a passive ergometric cycle and NMES (applied to one or both quadricep muscles). The duration of physical intervention varied between 20 min [30,32,33,34,35,39,40,42,44], 25 min [36], 30 min [37,38], and 50–60 min [29,43].

Early mobility protocols were mainly guided by daily mobilisation goals, following a stepwise approach from level 1 (no mobilisation) to level 5 (intensified therapy with activities of daily living) [30]. On the other hand, Winkelman’s “low-intensity” stage began with an in-bed range of motion (no resistance) or passive transfer to a chair (with no or very limited volitional muscle activation), advancing to higher levels of activation, or “moderate intensity” (sitting at the edge of bed, standing, pivot transfer to bedside chair, marching in place at bedside, or walking) [32].

Neuromuscular electrical stimulation intervention protocols varied widely among studies, being applied with an amplitude of 20–70 mA, symmetrical biphasic square waves with a frequency of 30–50 Hz, 6–12 s of contraction (“On” phase), 1.5–2 s of increase, 0.75 s of decrease, 6–25 s of rest (“Off” phase), and a pulse duration of 400 μs. The current amplitude was applied as high as possible to evoke maximum contractions in each muscle group [31,36,37,45]. Functional electrical stimulation used similar programming, differing in the intensity threshold, which was set to a maximum of 100 mA [38,39,40].

### 3.5. Myokine Sampling 

The most common methods for measuring serum [32,33,34,35,36,39,42,45] and plasma [30,31,33,37,40,44] myokine levels included commercially available colourimetric Enzyme-Linked ImmunoSorbent Assay (ELISA) kits (Berthold Technologies GmbH & Co.KG (Bad Wildbad, Germany), Zentech (Liège, Belgium), R-Biopharm (Darmstadt, Germany), and Neogen Corporation (Lansing, MI, USA)) [30,31,36,39,40,42,44,45], Meso-Scale Discovery Multiplex electrochemiluminescence kits (Rockville, MD, USA) [32,34,35], and Multiplex Luminex (xMAP technology, Waltham, MA, USA) assays [37,38]. Muscle biopsies were used to evaluate mRNA expression by sampling patients’ vastus lateralis [29,30,31] or rectus femoris [43], followed by quantitative analysis via real-time polymerase chain reaction. Myokines were typically measured at baseline after a period of rest and immediately after the cessation of intervention [34,35,45] or 10 min to 1 h after cessation [32,33,39,40,42,44]. In other cases, samples were collected on specific days of hospitalisation [29,30,31,36,37,38]. A detailed description of the methods used for myokine assessment can be found in Table 2. 

### 3.6. Overall Myokine Change

**Figure 2:**
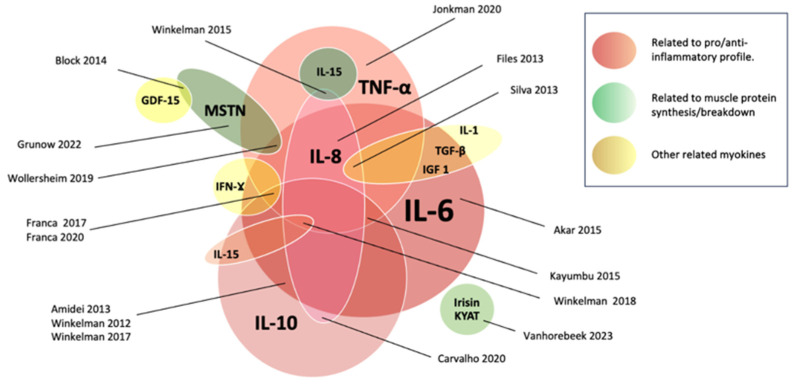
Myokines according to their role, interaction, and reported levels. Myokines related to the pro/anti-inflammatory role are represented by red tones. Those related to muscle protein metabolism are represented by green tones, and other myokines are represented by yellow. The sizes of the circles are associated with the number of times reported in the included studies [29,30,31,32,33,34,35,36,37,38,39,40,41,42,43,44,45].

The most common myokine reported was IL-6, which was included by 12 out of 17 studies (71%) [31,32,33,35,36,37,38,40,41,42,44,45]. This was followed by IL-10, which was studied by 11 out of 17 articles (65%) [32,33,35,36,37,38,39,40,42,44,45]; TNF-α, which was studies in 10 out of 17 studies (59%) [31,32,34,36,37,38,39,40,41,45]; IL-8 in 6 out of 17 (35%) [32,34,36,41,42,45]; and myostatin, which was cited in only 3 studies (18%) [30,31,43]. Additionally, IL-15 [32], IL-1 [36], and irisin [29], among other potential myokines (IFN-g; AA1/2, IGF-I, KYATs KYAT1/Kyat1 and KYAT3/Kyat3, and total amylase or Amy1) were reported [29]. The landscape of myokines reported by the authors can be found in Figure 2.

The analysed studies exhibited heterogeneous results concerning the plasmatic secretion or gene expression of myokines in response to physical intervention. Most (13 out of 17 studies; 77%) reported significant changes in myokine secretion or gene expression following intervention. Two of these studies reported a myokine reduction as the unique change [38,45]. The remaining studies reported a mixed pattern (increased, decreased, and no changes in a mixed manner) depending on the role of the myokine, the intervention used, and the considered outcome. Only 4 of the 17 included studies (24%) declared no significant change for any of the studied myokines [32,36,40,41].

### 3.7. Myokine Changes and Potential Clinical Effects According to Their Role

#### 3.7.1. Effects Related to Pro/Anti-Inflammatory Profile

After 20 sessions of NMES plus active joint motion exercise or just active exercise for both upper and lower extremities, Akar et al. [45] reported a significant decrease in serum IL-6 and IL-8 for those with both modalities combined and an IL-8 decrease for the group receiving only NMES. After the program, groups including NMES showed improvements in lower-extremity muscle strength, while upper-extremity muscle strength improved significantly in all groups. Similarly, after a single 20 min session of 20 flexion–extension movements per minute in each leg simultaneously, Amidei et al. [44] showed a significant decrease in IL-6 from baseline, without change after 60 min. The IL-6:IL-10 ratio significantly decreased at the end of the exercise and again after 60 min of rest. However, no significant difference was noted in IL-10 values at any time. Similarly, after passive exercise sessions, Winkelman et al. showed an improved IL-6:IL-10 ratio [33].

Despite the findings mentioned above, most studies reported no consistent change regarding IL-6 [34,37,39,40,42], with an exceptional increase in IL-6. The latter was the case for Wollersheim et al. [31], who used daily muscle-activating measures such as NMES and/or whole-body vibration (WBV) throughout the ICU stay—up to day 28—in addition to an early mobilisation protocol and only early protocolised mobilisation as a control group. Encoding genes for IL-6 and SAA1/2 (encoding for serum amyloid A1/2) both significantly increased the above values for healthy references, while TNF (encoding for tumour necrosis factor-alpha) presented values like those of healthy references for the intervention and control group. No association with clinical outcomes such as weaning success or length of stay was found.

Jonkman and colleagues [38] reported a between-group trend toward a decrease in TNF-α for the active group versus no change in the sham group. The first group consisted of patients receiving expiratory muscle FES during exhalation via surface electrodes on the abdominal wall (daily for 5 days per week until patients were weaned from MV). Additionally, they reported a change in total abdominal expiratory muscle thickness on day three in favour of the FES group.

#### 3.7.2. Effects Related to Muscle Protein Synthesis/Breakdown

In response to daily muscle-activating measures, as previously detailed, Wollersheim et al. found that encoding myostatin gene expression (MSTN) and myostatin plasma levels (a potent negative regulator of muscle mass [19]), generally associated with sarcopenia, were significantly decreased in the intervention group and remained unaffected by the type of intervention. ICU-AW occurred within the entire cohort, and muscle-activating measures did not improve muscle strength or function at the first awakening or 12-month follow-up. However, the myocyte cross-sectional area in the intervention group was significantly larger in comparison with the control group and the common physiotherapeutic group [31].

Block et al. [43] showed that after a single-leg, twice-a-day NMES session (one hour for one week), myostatin and GDF-15 mRNA expression was significantly elevated compared to baseline. Myostatin remained unchanged in the control leg.

After daily muscle-activating measures such as NMES (performed bilaterally on eight different muscle groups for 20 min) or whole-body vibration in addition to protocol-based physiotherapy, Grunow et al. [30] reported a significantly reduced MSTN in skeletal muscle when compared to healthy controls, regardless of the level of mobilisation (*p* = 0.004). Reduced myostatin plasma levels were observed during the first 2 weeks of the ICU stay (*p* < 0.001) and were pronounced during the early phase of ICU treatment but with a significant increase over time (*p* < 0.001). Low myostatin plasma concentrations on day 8 were also correlated with reduced muscle strength at first awakening, while no correlation was observed at ICU discharge.

In a retrospective analysis including data from 12 ICU patients involved in a previous RCT study who received physiotherapy, early mobilisation, and single-leg NMES (one hour daily for 7 consecutive days), Vanhorebeek et al. [29] reported increased FNDC5 mRNA expression (precursor of irisin, a myokine able to promote myogenic differentiation and myoblast fusion via activation of IL-6 signalling [46,47]) in the stimulated leg. They also reported that patients who acquired clinically relevant muscle weakness in the ICU exhibited greater FNDC5 expression than patients who did not develop such weakness, aligning with the finding that ICU non-survivors showed lower FNDC5 expression than ICU survivors in univariable analysis [29].

#### 3.7.3. Others

Vanhorebeek et al. [29] measured IFN-g, AA1/2, IGF-I, and KYATs KYAT1/Kyat1 and KYAT3/Kyat3, as well as total amylase or Amy1 (markers of peripheral metabolism able to elicit distant beneficial effects on energy utilisation in adipose tissue [29,48]), finding remarkably low vastus lateralis expressions of FNDC5, KYAT, and amylase mRNA after 1 week in the ICU compared to healthy subjects. This low FNDC5 expression was associated with a higher risk of death and weakness, while lower amylase expression had a more prolonged dependency on intensive care. Interestingly, lower amylase or KYAT1 expression was associated with a lower risk of death or weakness. NMES was able to increase FNDC5 expression compared with the unstimulated muscle but did not affect the other myokines [29].

After twice-a-day NMES sessions, Block et al. [43] showed that the Growth Differentiation Factor-15 (GDF-15, a myokine able to increase thermogenesis and improve insulin sensitivity [49,50]) mRNA expression was significantly elevated in NMES legs compared to baseline but remained unchanged in control legs.

A general scheme of reported myokines according to their role can be found in Figure 2.

### 3.8. Myokine Changes According to the Intervention Type

#### 3.8.1. Passive Mobilization (PM)

Three studies included passive exercise as the exclusive intervention [40,42,44]. In the quasi-experimental study of Amidei et al. [44], a 20 min PM session was applied to 30 patients, revealing significant decreases in IL-6 values from baseline to completion, with no difference observed 60 min later. No significant difference was noted in IL-10 values at any time. Similarly, Carvalho et al. [42], also in a quasi-experimental study involving 11 deeply sedated patients, applied a single 20 min session of passive cycle ergometer exercise. This showed a significant decrease in IL-8 after the 60 min session compared to baseline, while no significant changes were observed after the 20 min passive cycle ergometer session. IL-10, on the other hand, showed a significant increase immediately after the session and 60 min after it.

Franca et al. conducted an RCT with 19 patients, also applying a single PM session (cycle ergometry on lower limbs), finding no significant changes in any cytokine (TNF-α, IFN-g, IL-6, or IL-10 serum levels) [40].

#### 3.8.2. Early Mobilisation (EM)

Two studies reported mobilisation (referred to as rehabilitation) as the only intervention implemented [34,35]. However, to better understand the intervention, these articles, based on early rehabilitation, also considered PM as the initial step. Therefore, articles with rehabilitation protocols that include PM (as an initial step), such as studies including unconscious patients, are also considered in this section.

Files et al. randomly treated 50 ICU patients with EM and 50 patients with usual care. The former consisted of two physical therapy sessions per day for 7 days and resistance training. Through day 7, TNF-α, IL-6, and IL-8 did not differ between groups, despite similar baseline acuity and inflammatory profiles [41].

In 2012, Winkelman et al. [35] compared patients receiving EM with those receiving standard care. The first group received 20 min (for 2–7 days) of in-bed exercise or initiated out-of-bed exercise. No significant associations between change in IL-6 and either mode or duration of exercise were found. There was a statistically significant association between change in IL-10 and exercise duration: the more significant the exercise duration, the lower the IL-10. However, the modality of exercise was not associated with changes in IL-10. In 2015, the same authors [44] used a similar progressive EM protocol, starting in unconscious patients with PM and combining an active/passive range of motion and transfers for conscious patients. TNF-α values were significantly associated with the type of activity (i.e., in bed vs. sitting at the edge of the bed or out of bed). Low TNF-α values in patients were associated with out-of-bed activity; conversely, high TNF-α values were related to in-bed activity.

In 2007, using a similar modality, Winkelman et al. reported that the average ratio of IL-6 to IL-10 improved after an average of 14.7 min of PM (51.4 (17.3) pg/mL at rest and 41.0 (13.2) pg/mL after activity. Significant differences between average resting and activity values of IL-6 (*p* < 0.001) but not of IL-10 were found [33].

Finally, in 2018, the same author delivered an EM intervention to two groups: “low” and “moderate” intensity. “Low” was offered as an in-bed range of motion or passive transfer to a chair, with no or very limited volitional muscle activation. “Moderate” involved sitting at the bed’s edge, standing, pivoting to the bedside chair, marching in place at the bedside, or walking. There were no significant differences in baseline values for any of the inflammatory biomarkers, nor any association with the intensity of EM [32].

#### 3.8.3. NMES/FES

Only two studies isolated electrical currents for muscle stimulation, while the rest applied them in combination with other exercise modalities. Block et al. applied NMES twice a day for one hour for one week [43]. They found that myostatin and GDF-15 mRNA expressions were significantly elevated in NMES legs compared to baseline but remained unchanged in control legs.

Jonkman et al. [38] applied expiratory muscle FES during exhalation via surface electrodes on the abdominal wall. There was a between-group difference for TNF-α, with a trend towards a decrease for the active group versus no change in the sham group.

## 4. Discussion

This scoping review introduces the intricate landscape of myokine secretion and its local or potential systemic effects in critically ill patients, particularly in the early stages of the disease. Notably, most included studies were published within the last decade, indicating a growing interest in this field. We identified a broad spectrum of study designs, which limits their interpretation. Observational or quasi-experimental studies do not allow us to establish causality, and although we identified eight RCTs, the number of participants recruited confirms that we still know little about myokine dynamics in critically ill patients. Furthermore, the heterogeneity of the interventions applied in the studies that met the eligibility criteria of our scoping review, in terms of their type and dosage, makes it challenging to identify any pattern in the behaviour of myokine secretion and concentration. Some studies explored acute responses to physical function rehabilitation after a single session; others included prolonged rehabilitation programs, and not all interventions aimed to achieve voluntary contraction of the participants’ muscles, adding more edges to this intricate topic. And if that were not enough, the methods of measuring myokine concentrations varied across the included studies.

Among the 17 included studies, disparities in patient characteristics, unit types, and vital support mechanisms were observed, which are also critical to consider regarding interpretation of the results. For instance, some studies focused on patients within 48–72 h from intubation and MV initiation, while others explored scenarios involving chronic critically ill patients with prolonged MV. Contextual differences are crucial, as they influence muscle responses to exercise, with confounding factors affecting the interpretation of relative changes in myokine secretion due to inflammatory contexts.

Myokine liberation in critically ill patients encompasses a spectrum of markers, including IL-6, IL-10, TNF-α, myostatin, IL-15, IL-1, and irisin. Cytokines, particularly IL-6, have been proposed as the original myokine, echoing ancient connections to exercise. However, the precise modality inducing myokine changes remains elusive, whether through passive, active, or physical stimuli like electrical currents. Myokine levels are even more challenging to interpret when considering the potential for a biomarker storm in critically ill patients. Although a significant source of bioactive molecules, muscle is not the only contributor, with numerous unidentified alterations potentially impacting cytokine release in pathological scenarios.

Examining the impact of interventions on pro/anti-inflammatory profiles and muscle protein synthesis/breakdown in critically ill patients revealed intriguing findings. Combined exercise modalities involving NMES and active joint motion exercise significantly decreased serum IL-6 and IL-8 levels. Passive exercise and simulated slow walking showed similar reductions in IL-6, while passive exercise specifically improved the IL-6:IL-10 ratio. TNF-α levels exhibited varying trends across interventions, with daily measures, including NMES, resulting in increased TNF-α gene expression. Concerning muscle protein synthesis and breakdown, daily muscle-activating measures significantly decreased myostatin gene expression and plasma levels. NMES sessions notably elevated myostatin and GDF-15 mRNA expression, and exercise-induced myokine irisin exhibited increased expression with NMES, emphasising its role in muscle health.

The uncertainty surrounding myostatin’s role in muscle weakness and atrophy during critical illness is further complicated. Grunow et al. showed no significant association between myostatin plasma concentrations and markers of mobilisation, electrophysiological variables, or atrophy pathways [30]. Myostatin may not be considered a key driver of muscle wasting in critically ill patients [47]. In this respect, it has been suggested that decorin could act as a modulator of myostatin activity by competitively binding to it, potentially preventing myostatin from exerting its inhibitory effects on muscle growth [19,51]. Questions about their complex interplay and actual role in muscle preservation should be retained for future investigations.

Several factors should be considered when interpreting the results of the included studies. The choice of myokines for assessment, the timing of myokine measurements, and the specific context of the ICU environment all influence the results. The elevated proteasomal pathway in critically ill patients introduces unidentified alterations in the muscle’s capacity to release myokines. This interplay of factors contributes to the challenges in comprehending myokine dynamics in critical illness, urging further research to unravel these complexities and inform targeted interventions with systemic impacts.

## 5. Conclusions

The comprehensive exploration of myokine dynamics in critically ill patients underscores the complexity of this emerging field. Studies conducted over the last decade indicate a growing interest and potential for research development. However, challenges related to study design, small sample sizes, and variations in intervention specifics across clinical trials contribute to the intricate nature of myokine responses. While progress has been made, challenges persist in selecting suitable myokines for analysis and determining optimal measurement timing. Gaps in understanding transient myokine changes in the ultra-acute phase and the impact of unidentified alterations in the muscle’s capacity to release myokines underscore the need for continued research. In essence, the evolving landscape of myokine research in critical illness calls for a comprehensive approach, considering the countless factors influencing myokine responses, to inform targeted interventions and improve patient outcomes in the ICU.

To overcome the remaining challenges, future studies should prioritise understanding myokine release patterns, determining suitable biomarkers sensitive enough to be adequate in the critically ill context, and determining and standardizing the timing and type of interventions and measurements for myokine studies.

## Figures and Tables

**Table 1:** Search strategy for MEDLINE (Ovid).

N°	Search Term
1	exp Cytokines/
2	exp Chemokines/
3	exp Interleukins/
4	exp Biomarkers/
5	(cytokin$ or chemokin$ or interleukin$ or miokin$ or exerkin$ or biomarker$ or biologic$ marker$).mp,tw.
6	(Angiopoietin-like 4 or ANGPTL4 or Apelin or aminoisobutyric acid or BAIBA or Brain-derived neurotrophic factor or BDNF or Chemokine ligand or C-X-C motif or Decorin or Fibroblast growth factor 21 or FGF21 or IL-6 or IL-8 or IL-10 or IL-13 or IL-15 or IL-18 or Irisin or FNDC5 or Musclin or Myonectin or C1QTNF5 or Myostatin or Leukemia inhibitory factor or LIF or Secreted protein acidic rich in cysteine or SPARC or Tumor necrosis factor$ or TNF$).mp,tw.
7	or/1–6
8	exp intensive Care/
9	exp critical Illness/
10	exp Critical Care/
11	exp Intensive Care Units/
12	((intensive or critic$) adj2 (care or ill$)).mp,tw.
13	exp Respiration, Artificial/
14	exp Ventilator Weaning/
15	((mechanic$ or artificial) adj2 (ventilat$ or respirat$)).mp,tw.
16	or/8–15
17	exp Electric Stimulation Therapy/
18	exp Transcutaneous Electric Nerve Stimulation/
19	exp Electric Stimulation/
20	((neuromusc$ or functional or transcutan$) adj2 (electric$ or electrotherap$)).mp,tw.
21	(electrotherap$ or electromyostimulation or electrostimulation or (electric$ adj2 stimulation)).mp,tw.
22	(NMES or FES or TENS).mp,tw.
23	or/17–22
24	exp Exercise/
25	exp Exercise Therapy/
26	exp Physical Therapy Modalities/
27	exp Occupational Therapy/
28	exp Muscle Contraction/
29	exp Resistance Training/
30	(physiotherap$ or physical therap$ or kinesiotherap$ or physical rehabilitation or exercis$ or train$).mp,tw.
31	(musc$ adj2 contrac$).mp,tw.
32	or/24–31
33	or/23,32
34	and/7,16,33

**Table 2:** Protocols and results of included studies.

Author/	Population	Intervention	Program	Biomarkers	Method of Evaluation	Results	Clinical Impact
Year/							
Country/Study Design							
Akar et al., 2017 [45] (Turkey) RCT	COPD-conscious patients in early intubation period.	Group 1: AT exercise for upper and lower extremities and NMES (amplitude of 20–25 mA, symmetrical biphasic square waves with 6 s of contraction, 1.5 s of increase and 0.75 s of decrease, and wave frequency of 50 Hz). Group 2: only NMES. Group 3: only AT.	5 days per week for a total of 20 sessions.	IL-6, IL-8, IL-10, and TNF-α	Blood samples were taken twice: once before the intervention, then at the end of the intervention. Serum biomarker levels were measured using commercially prepared ELISA kits.	For group 1, serum IL-6 significantly decreased after the program (*p* = 0.015), and significant decreases were also noted in serum IL-8 for groups 1 and 2 (*p* = 0.017).	After the program, lower-extremity muscle strength improved significantly in groups 1 and 2, while upper-extremity muscle strength improved significantly in all groups. The weaning time of the groups was comparable. Time to sit up assisted/unassisted in bed and at the bedside and the time to move from bed to chair were similar for all groups.
Amidei et al., 2013 [44] (United States). Quasi-experimental	Critically ill adults within 48 h of intubation and MV use.	PM: 20 min of 20 flexion–extension (from 70° flexion to 5° flexion) movements per minute in each leg simultaneously. Leg movements were alternated, simulating slow walking.	Single session.	IL-6 and IL-10	Blood samples were taken at three-time points: baseline, upon intervention completion, and one hour after. Plasmatic biomarker levels were measured via commercially prepared ELISA kits.	A significant decrease in IL-6 values was found from baseline to the completion of the exercise (*p* = 0.03), but there was no difference 60 min later. No significant difference was noted in IL-10 values at any time. The IL-6:IL-10 ratios showed a significant decrease at the end of the exercise and again after 60 min of rest (*p* = 0.05).	At all study points, heart rates and mean systolic blood pressures were in normal ranges for physiological status. The Body Pain Score showed a significant decrease at all points.
Bloch et al., 2014 [43] (United Kingdom) Contralateral controlled trial	Patients admitted to a cardiothoracic ICU recruited before elective high-risk cardiac surgery or during ICU admission.	NMES performed twice a day for 1 h in one leg.	Every day for one week.	Myostatin and GDF-15	Rectus femoris biopsies were taken before and after intervention (no additional information about sample processing was provided).	Myostatin and GDF-15 mRNA expressions were significantly elevated in NMES legs compared to baseline (*p* = 0.03 and *p* = 0.04, respectively) but remained unchanged in control legs.	There was no significant change in the bilateral rectus femoris cross-sectional area measured by ultrasound.
Carvalho et al., 2020 [42] (Brazil) Prospective study	Mechanically ventilated patients within 48 h of intubation—haemodynamically stable with deep sedation.	20 consecutive minutes of PM using EC at a fixed pedalling rate of 20 cycles/minute (20 min).	Single session.	IL-8 and IL-10	Blood samples were taken at three time points: before the intervention, immediately after the intervention, and one hour after it. Serum biomarker levels were assessed using commercially prepared ELISA kits.	IL-8 decreased significantly 1 h after the session compared to baseline (*p* = 0.001), but there were no significant changes after the 20 min-session. Similarly, IL-10 showed a significant increase immediately after the end of the session and 60 min after (*p* < 0.001).	NR
Files et al., 2013 [41] (United States) RCT	Acute respiratory failure ICU patients.	ER: two physical therapy sessions per day, with the second session comprising resistance training. UC: one session.	Every day for one week.	TNF-α, IL-6, and IL-8	Blood was drawn for cytokines through day 7 (no additional information about sample processing was provided).	Biomarkers through day 7 were did not differ between groups despite similar baseline acuity and inflammatory profiles.	There was no significant change in grip strength, dynamometry, or Short Physical Performance (SPPB). However, the latter showed a minimal clinically significant difference.
Franca et al., 2017 [40] (Brazil) RCT	Mechanically ventilated patients.	Group 1: PM through EC on lower limbs with speed adjusted to 30 cycles per minute for 20 min. Group 2: Control, without intervention.	Single session.	L-6, IL-10, IFN-γ, and TNF-α	Blood samples were taken twice: once before intervention, then one hour after intervention. Samples were stored in collection tubes containing dipotassium ethylenediamine tetra-acetic acid. Plasmatic levels were assessed using commercially prepared ELISA kits.	No significant changes in serum levels before and after for the two groups.	NR
Franca et al., 2020 [39] (Brazil) RCT	Patients admitted to ICU.	Group 1: Control. Group 2: PM through EC with 30 cycles per minute for 20 min. Group 3: FES (500 ms pulse width, 50 Hz, 2 s of elevation, 5 s of support, and 2 s of descent with a 1:1 ON/OFF time; 20 min for each stimulated quadricep muscle). Group 4: EC and FES.	Single session.	IL-6, IL-10, IFN-γ, and TNF-α	Blood samples were taken twice: once before intervention, then one hour after intervention. Samples were stored in collection tubes containing dipotassium ethylenediamine tetra-acetic acid. Then, they were diluted in 10 mL falcon tubes for nitrosative stress analysis, determining nitric oxide production in cells and assessing serum biomarkers using commercially prepared ELISA kits.	IFN-γ, IL-6, and IL-10 levels did not significantly decrease before and after the intervention. TNF-α levels were reduced after the PM intervention (*p* = 0.049).	NR
Jonkman et al., 2020 [38] (Netherlands) Prospective study (feasibility study)	Mixed ICU population.	FES: For external and internal oblique and transversus abdominis muscles [30 Hz, pulse width of 352 μs, and maximum intensity of 100 mA (according to visual muscle contraction)]. Control: Sham NMES without muscle contraction (frequency of 10 Hz, pulse width of 352 μs, and intensity of 10 mA).	FES was applied for 30 min, twice daily, five days per week until patients were weaned from mechanical ventilation—but no longer than six weeks.	TNF-α, IL-1 receptor antagonist, IL-1β, IL-6, IL-8, and IL-10	Blood samples were collected at baseline and on day 3 for plasmatic biomarker analysis using a Multiplex Luminex assay	There was a between-group difference in the change in pro-inflammatory marker TNF-α, with a trend toward a decrease for the active group versus no change in the sham group.	The total abdominal expiratory muscle thickness changed on day 3, favouring the FES group. No difference was found on day 5 or over days. Using a survival analysis approach, median ventilation duration and ICU length of stay were shorter for the FES group (*p*-values of 0.07 and 0.03, respectively). No difference was observed between median mechanical ventilation use and median ICU length of stay.
Kayambu et al., 2015 [27] (Australia) Prospective double-blinded RCT	Septic patients admitted to ICU.	ER: Individualised early physical rehabilitation strategies including NMES (vastus tedialis, vastus lateralis, tibialis anterior, and brachioradialis; 40–45 Hz at 20–25 mA, pulse width of 400 μs (12 s on and 6 s off)), PM, AT, sitting out of bed, transfers, ambulation, and other mobilisation techniques. Control: standard care.	ER program for 30 min, once or twice daily, until discharge from the ICU and within 48 h of the diagnosis of sepsis.	IL-6, IL-10, and TNF-α	Blood samples were collected on days 1, 3, 5, and 7 after the trial’s initiation and after ICU discharge. Biomarkers were measured from plasma samples using Milliplex cytokine panels from Millipore and a Luminex 100 assay.	A reduction in mean IL-6 scores was observed in the ER group pre and post exercise across days 1, 3, 5, and 7, but no statistically significant differences were found between groups. Overall, the mean change in IL-10 from baseline to ICU discharge was significantly higher for the ER group (*p* = 0.04). For TNF-α, there was a significant decrease between groups from baseline to ICU discharge (*p* < 0.01).	No differences were found in the physical function and mobility scores. In the ER group, self-reported quality of life improved in the domains of physical function and physical role compared to those receiving standard care. No differences were found in ICU exercise capacity (PFIT score) or MRC score.
Silva et al., 2019 [36] (Brazil) RCT	Critically ill traumatic brain injury (TBI) patients.	NMES group: ER plus NMES once a day (400 μs and 100 Hz with 5 s on and 25 s off, eliciting 50 contractions per day. The current intensity was titrated to evoke maximum contractions in the quadriceps, hamstring, tibialis anterior, and gastrocnemius). Control: ER including PM, AT, sitting out of bed, transfers, and ambulation.	NMES was applied daily (one time) for 25 min for 14 days. ER was applied for 10–30 min.	IGF-1, TGF-β, IL-1β, IL-6, IL-8, IL-10, and TNF-α	Blood samples were collected 24 h after admission and on days 3, 7, and 14. Serum levels of IGF-1 and TGF-β were measured using commercially prepared ELISA kits, while circulating concentrations of IL-1β, IL-6, IL-8, IL-10, and TNF-α were assessed through a multiplex flow cytometry technique.	Plasma IGF-1, TGF-β, and IL-10 presented a statistically significant time–effect interaction (*p* = 0.01, 0.03, and 0.05, respectively). Meanwhile, TNF-α, IL-1 β, IL-6, and IL-8 did not present differences regarding group/intervention interaction.	The control group showed a significant reduction in muscle thickness of the tibialis anterior and rectus femoris, while it was preserved in the NMES group. The control group also had a higher incidence of neuromuscular electrophysiological disorders. NMES determined an increase in the evoked peak force versus the control group.
Winkelman et al., 2007 [33] (United States) Prospective correlational and exploratory	Patients receiving MV for more than 3 days and with an ICU stay of 5 to 15 days.	AT: Baseline activity or all movement initiated by participants. ER: Therapeutic activity, both active (i.e., patient-initiated) and passive (i.e., initiated and supported by a healthcare worker). All therapeutic motions were recorded.	Minimum duration of 10 min for PM. Data and the type and duration of activity and PM were collected within one 24-h period (two separate 4-h blocks).	IL-6 and IL-10	Blood samples were taken twice under a 24 h margin: once after a period of 10 min of activity, then immediately after a period of 60 min of uninterrupted rest. The serum was analysed using commercially available antibodies, with duplicate readings for each cytokine. Then, after adding an enzyme-conjugated polyclonal detection antibody, the colourimetric substrate was assessed by a single experienced technician.	The average ratio of IL-6 to IL-10 improved after 14.7 min of passive physical activity. Significant differences existed between the average resting and activity values of IL-6 (*p* < 0.001) but not for IL-10.	IL-6 and IL-10 and their ratio were not associated with patient outcomes of weaning success or length of stay. High levels of IL-6 were associated with mortality.
Winkelman et al., 2012 [35] (United States) Prospective, exploratory, repeated measures interventional study	Subjects admitted to a medical or surgical ICU.	Control: No exercise was planned, nor did participants routinely receive exercise from a physical therapist. ER: In-bed exercise or initiated out-of-bed exercise based on the protocol’s criteria (ability to follow three out of five directions, lift both arms off the bed, and/or lift each leg off the bed).	ER was provided for 20 min once daily for 2–7 days.	IL-6 and IL-10	Blood samples were taken twice daily on 3 contiguous study days after enrolment and again on day 7 after enrolment: once after a period of 30 min of rest, then immediately after cessation of the intervention to assess serum biomarker levels using a meso-scale discovery multiplex antibody electrochemiluminescence approach.	After controlling for resting IL-6, age, group, gender, race, BMI, and severity, there were no associations between IL-6 and either mode or duration of exercise. However, a significant association between IL-10 and exercise duration was found: the more substantial the duration, the lower the IL-10 during both control and intervention (*p* = 0.03). The mode of exercise was not associated with changes in IL-10. Change in IL-6 was associated with self-reported fatigue but not pain (*p* = 0.02).	Participants enrolled in the intervention period experienced 5 fewer days of ICU hospitalisation than the control group, despite higher acuity on ICU admission. The duration of mechanical ventilation was not different between groups.
Winkelman et al., 2015 [34] (United States) Prospective, exploratory, repeated-measures interventional study.	Patients receiving MV for > 48 h with no plan to extubate in the subsequent 48 h.	ER: All participants received a protocolised activity. Level 1: unconscious patients receiving PM; level 2: conscious patients receiving combined active/PM; level 3: weight-bearing transfer to a chair, advancing to dangling; level 4: advancing to standing and ambulation.	ER was provided for 20 min once daily for three days.	IL-8, IL-15, and TNF-α	Blood samples were taken twice daily on 3 contiguous study days: once after a period of at least 30 min of rest, then immediately after cessation of the intervention to assess serum biomarker levels using a meso-scale discovery multiplex anti-body electrochemiluminescence approach.	No significance for IL-8, IL-15, and TNF-α was found in terms of within-subject variations. No association with ICU severity of illness (APACHE 3) on admission was observed. TNF-α values were significantly associated with the type of activity (in bed vs. out of bed). Low TNF-α values were related to out-of-bed activity, and high TNF-α values were associated with in-bed activity (*p* = 0.02).	Among the 23 participants in whom muscle strength was measured, 8 (35%) scored < 24 on the MMT and values consistent with ICUAW. Among the 32 participants in whom we measured ADLs, 24 (75%) were moderately to severely dependent on ADLs (i.e., KATZ score < 4) the day after ICU discharge.
Winkelman et al., 2018 [32] (United States) RCT study with repeated measures and blinded assessment of outcomes	Patients receiving MV for 36 h and study expected to require 24 h more.	Low ER: In-bed range of motion (no resistance) or passive transfer to a chair, providing full support. Moderate ER: Sitting at the edge of the bed, standing, pivoting transfer to bedside chair, marching in place at the bedside, or walking.	ETM was sustained daily until ICU discharge. The comparison group comprised patients receiving one or two daily ER sessions.	IL-6, IL-8, IL-10, IL-15, and TNF-α	Blood samples were taken twice daily on 3 contiguous study days: once after a period of rest, then 20 min after cessation of the intervention to assess serum biomarker levels using a meso-scale discovery multiplex antibody electrochemiluminescence approach	There were no significant differences in baseline values for any biomarker comparing once- or twice-a-day sessions at any intensity. There was no significant association of inflammatory biomarkers with the intensity of ER.	ICU LOS was significantly reduced for patients who received ER sessions twice daily compared to once. MMT scores and dynamometer handgrip values were more outstanding on days 1 and 3 among participants who received moderate-intensity ER than those receiving low ER. Similarly, reduced delirium on days 1 and 3 was found for participants receiving moderate-intensity ER.
Wollersheim et al., 2019 [31] (Germany) Exploratory RCT single-centre trial	Mechanically ventilated patients with sepsis-related MODS (SOFA score ≥ 9 within the first 72 h after ICU admission).	Control: Common ER performed. ER+ muscle-activating measures: The intervention group added NMES or WBV to the ER. NMES was performed bilaterally on eight different muscles, starting on enrolment day (maximum intensity of 70 mA until visible/palpable muscle contraction). WBV used 20 cycles (alternating stimulation, 26 Hz, and amplitude of 15 mm), with 1 min pause following each 1 min stimulation cycle. Common PT: PT performed without a clear protocol.	Common PT was performed only on weekdays. ER, NMES, and whole-body vibration (WBV) were carried out daily throughout the ICU and lasted until day 28, in addition to protocol-based physiotherapy. NMES lasted 20 min.	Myostatin, MSTN, IL6, TNF-α, and SAA1/2	Blood samples were taken on day 14 after ICU admission to assess myostatin plasma levels using commercially prepared ELISA kits. Additionally, on day 15, muscle biopsies were sampled from the patient’s vastus lateralis to quantify gene expression via real-time polymerase chain reactions and protein concentration via western blot analyses.	MSTN and myostatin plasma levels significantly decreased in both groups, and the patients remained unaffected by the intervention. IL-6 and SAA1/2 significantly increased the above values for healthy references, while TNF-α was like healthy references for the intervention and control groups, without differences between these two groups. Comparing standard PT with both groups, a significantly increased gene expression for TNF-α (*p* < 0.001) and a considerable decrease in SAA1/2 (*p* = 0.008) were found. TNF expression was increased in standard PT in contrast to healthy values.	ICU-AW occurred within the entire cohort, and muscle-activating measures did not improve muscle strength or function at first awakening or 12-month follow-up. No signs of necrosis or inflammatory infiltration were present in the histological analysis. The myocyte cross-sectional area in the intervention group was significantly larger in comparison with the control group (type I, +10%; type IIa, +13%; type IIb, +3%) and CPP (type I, +36%; type IIa, +49%; type IIb, +65%).
Vanhorebeek et al., 2023 [29] (Belgium) Retrospective analysis (data from two RCTs).	Participants in the Early Parenteral Nutrition Completing Enteral Nutrition in Adult Critically Ill Patients (EPaNIC RCT) study were included. The second study included patients who participated in the NMES in Critically Ill Patients (NESCI) study.	In the EPaNIC study, standard PT was provided to both groups (with and without nutritional intervention). In the NESCI study, adult patients between days 2 and 4 after ICU admission received PT and ER according to a standardised local “Start to move as soon as possible” protocol. Randomization determined whether the quadriceps of the dominant or the nondominant leg were selected for stimulation.	PT and ER were provided daily according to unit protocols. The NMES session lasted for 1 h daily for 7 consecutive days (50 min at the highest tolerable intensity with a visible muscle response; 8.5 s ON, 45 Hz, pulse of 350 μs, 1.5 s ramp up, 1 s ramp down, and 12-s OFF phase).	FNDC5, KYAT1, KYAT3, AMY1A, AMY1B, AMY1C, AMY2A, and AMY2B	Muscle biopsies were taken on day 8±1 after ICU admission. The de Bergström technique was used on the patient’s vastus lateralis to assess mRNA expression. mRNA extraction was performed using an in-house protocol. Quantification was performed via real-time polymerase chain reaction.	In critically ill patients, mRNA expression of FNDC5, KYAT1, and total amylases in muscle was 34% to 80% lower than in controls, whereas no significant effect was observed for KYAT3. NMES that resulted in good muscle contraction increased FNDC5 expression compared with unstimulated muscle but did not affect the other myokines.	In univariable analysis, ICU non-survivors showed lower FNDC5 expression than ICU survivors. In multivariable analysis, lower FNDC5 expression remained independently associated with a higher risk of death in the ICU. ICU survivors who needed more than 7 days of intensive care after collecting the muscle biopsy had lower expressions of FNDC5 and amylases than ICU survivors who needed a shorter ICU stay. Patients who acquired clinically relevant muscle weakness in the ICU had a lower expression of FNDC5 than patients who did not develop such weakness.
Grunow and cols, 2022 [30] (Germany) Retrospective cohort analysis (data from two prospective clinical studies)	Patients in both studies fulfilled the same inclusion criteria: age > 18, MV, and high risk for developing ICU-AW (SOFA score ≥ 9 within the first 72 h after ICU admission).	Common PT (sPT) or PT guided by daily mobilisation goals (pPT) provided by a stepwise approach. Level 1 (no mobilization) was used until level 5 (intensified therapy with activities of daily living) or pPT+ muscle activating measures (NMES and/or WBV).	For 14–15 days, muscle-activating measures were carried out daily throughout the ICU stay up to day 28, in addition to protocol-based physiotherapy. NMES was performed bilaterally on eight different muscle groups for 20 min. WBV was performed daily for 20 cycles.	Myostatin and MSTN	Blood samples were collected on days 4, 8, and 14 using commercially prepared ELISA kits to determine myostatin plasma levels. Additionally, muscle biopsies were sampled from the patient’s vastus lateralis on day 15 after ICU admission to isolate mRNA expression of MSTN via TRIzol reagent, then quantified by real-time quantitative polymerase chain reaction.	Patients were categorised into two subgroups on day 14 according to myostatin plasma levels: increased/decreased levels when compared to healthy controls. Critically ill patients showed significantly reduced MSTN gene expression in skeletal muscle compared to healthy controls (*p* = 0.004). Reduced myostatin plasma levels were observed during the first 2 weeks of the ICU stay (*p* < 0.001) and were pronounced during the early phase of ICU treatment but with a significant increase over time (*p* < 0.001).	Muscular MSTN gene expression on day 15 after ICU admission was significantly lower in ICU-AW patients than in controls and patients without ICU-AW. Low myostatin plasma concentrations on day 8 correlated with reduced muscle strength at first awakening, while no correlation was observed at ICU discharge. MSTN gene expression decreased in all critically ill patients.

RCT: randomized clinical trial; AT: active joint motion; NMES: neuromuscular electrical stimulation; ER: early rehabilitation; PM: passive motion; EC: ergometric cycling; MRC: medical research council; ADL: daily life activities; MMT: manual muscle testing; LOS: length of stay; MODS: multiple-organ dysfunction syndrome; WBV: whole-body vibration; PT: physical therapy; ICU-AW: intensive care unit-acquired weakness; MV: mechanical ventilation; NR: not reported.

## Data Availability

All data generated or analysed during this study are included in this published article and its Supplementary Information files.

## Supplementary Materials

The following supporting information can be downloaded at: https://www.mdpi.com/article/10.3390/jcm14092892/s1, Table S1: search strategy; Table S2: reasons for exclusion of evaluated full-text studies.

## Abbreviations

The following abbreviations are used in this manuscript:

ICU	Intensive Care Unit
ICU-AW	Intensive Care Unit-Acquired Weakness
MV	Mechanical Ventilation
NMES	Neuromuscular Electrical Stimulation
JBI	Joanna Briggs Institute
INPLASY	International Platform of Registered Systematic Review and Meta-analysis Protocols
PRISMA-ScR	Preferred Reporting Items for Systematic reviews and Meta-Analyses extension for Scoping Reviews
CINAHL	Cumulated Index to Nursing and Allied Health Literature
RCT	Randomized Controlled Trial
COPD	Chronic Obstructive Pulmonary Disease
Hz	Hertz
μs	Microseconds
mA	Milliamperes
ELISA	Enzyme-Linked ImmunoSorbent Assay
mRNA	Messenger Ribonucleic Acid
IL-6	Interleukin 6
IL-10	Interleukin 10
TNF-α	Tumour Necrosis Factor α
IL-15	Interleukin 15
IL-1	Interleukin 1
IFN-g	Interferon Gamma
AA1/2	Serum Amyloid a1/2
IGF-I	Insulin-like Growth Factor
KYAT	Kynurenine-oxoglutarate Transaminase
Amy1	Total amylase
WBV	Whole body vibration
FES	Functional Electrical Stimulation
MSTN	Myostatin gene expression
GDF-15	Growth Differentiation Factor 15
FNDC5	Fibronenctin type III Domain-Containing protein 5
PM	Passive Mobilization
EM	Early Mobilization

## References

[1] Schreiber A., Bertoni M., Goligher E.C. (2018). Avoiding Respiratory and Peripheral Muscle Injury During Mechanical Ventilation: Diaphragm-Protective Ventilation and Early Mobilization. Crit. Care Clin..

[2] Kress J.P., Hall J.B. (2014). ICU-acquired weakness and recovery from critical illness. N. Engl. J. Med..

[3] Puthucheary Z.A., Rawal J., McPhail M., Connolly B., Ratnayake G., Chan P., Hopkinson N.S., Phadke R., Dew T., Sidhu P.S. (2013). Acute skeletal muscle wasting in critical illness. JAMA.

[4] Iwashyna T., Ely W., Smith D., Langa K.M. (2010). Long-term Cognitive Impairment and Functional Disability Among Survivors of Severe Sepsis. JAMA.

[5] Herridge M.S., Tansey C.M., Matté A., Tomlinson G., Diaz-Granados N., Cooper A., Guest C.B., Mazer C.D., Mehta S., Stewart T.E. (2011). Functional Diasability 5 years after Acute Respiratory Distress Syndrome. N. Engl. J. Med..

[6] Dres M., Goligher E.C., Heunks L.M.A., Brochard L.J. (2017). Critical illness-associated diaphragm weakness. Intensive Care Med..

[7] Van Der Schaaf M., Beelen A., Dongelmans D.A., Vroom M.B., Nollet F. (2009). Poor functional recovery after a critical illness: A longitudinal study. J. Rehabil. Med..

[8] Gruther W., Benesch T., Zorn C., Paternostro-Sluga T., Quittan M., Fialka-Moser V., Spiss C., Kainberger F., Crevenna R. (2008). Muscle wasting in intensive care patients: Ultrasound observation of the M. quadriceps femoris muscle layer. J. Rehabil. Med..

[9] Levine S., Nguyen T., Taylor N., Friscia M.E., Budak M.T., Rothenberg P., Zhu J., Sachdeva R., Sonnad S., Kaiser L.R. (2008). Rapid Disuse Atrophy of Diaphragm Fibers in Mechanically Ventilated Humans. N. Engl. J. Med..

[10] Goligher E.C., Dres M., Fan E., Rubenfeld G.D., Scales D.C., Herridge M.S., Vorona S., Sklar M.C., Rittayamai N., Lanys A. (2018). Mechanical ventilation-induced diaphragm atrophy strongly impacts clinical outcomes. Am. J. Respir. Crit. Care Med..

[11] Goligher E.C., Fan E., Herridge M.S., Murray A., Vorona S., Brace D., Rittayamai N., Lanys A., Tomlinson G., Singh J.M. (2015). Evolution of diaphragm thickness during mechanical ventilation: Impact of inspiratory effort. Am. J. Respir. Crit. Care Med..

[12] Zayed Y., Kheiri B., Barbarawi M., Chahine A., Rashdan L., Chintalapati S., Bachuwa G., Al-Sanouri I. (2020). Effects of neuromuscular electrical stimulation in critically ill patients: A systematic review and meta-analysis of randomised controlled trials. Aust. Crit. Care..

[13] Zanotti E., Felicetti G., Maini M., Fracchia C. (2003). Peripheral muscle strength training in bed-bound patients with COPD receiving mechanical ventilation: Effect of electrical stimulation. Chest.

[14] Langer D., Troosters T., Hermans G., Decramer M. (2009). Early exercise in critically ill patients enhances short-term functional recovery*. Crit. Care Med..

[15] Machado A dos S., Pires-Neto R.C., Carvalho M.T.X., Soares J.C., Cardoso D.M., de Albuquerque I.M. (2017). Efeito do exercício passivo em cicloergômetro na força muscular, tempo de ventilação mecânica e internação hospitalar em pacientes críticos: Ensaio clínico randomizado. J. Bras. Pneumol..

[16] Hickmann C.E., Laterre P.-F., Roeseler J., Castanares-Zapatero D. (2020). Rôle de l’exercice précoce dans la régulation de l’inflammation chez le patient critique. Médecine Intensive Réanimation.

[17] Pedersen B.K., Steensberg A., Fischer C., Keller C., Keller P., Plomgaard P., Febbraio M., Saltin B. (2003). Searching for the exercise factor: Is IL-6 a candidate?. J. Muscle Res. Cell Motil..

[18] Piccirillo R. (2019). Exercise-induced myokines with therapeutic potential for muscle wasting. Front. Physiol..

[19] Lightfoot A.P., Cooper R.G. (2016). The role of myokines in muscle health and disease. Curr. Opin. Rheumatol..

[20] Lee J.H., Jun H.S. (2019). Role of myokines in regulating skeletal muscle mass and function. Front. Physiol..

[21] Görgens S.W., Eckardt K., Jensen J., Drevon C.A., Eckel J. (2015). Exercise and Regulation of Adipokine and Myokine Production. Prog. Mol. Biol. Transl. Sci..

[22] Wageck B., Nunes G.S., Silva F.L., Damasceno M.C.P., de Noronha M. (2014). Application and effects of neuromuscular electrical stimulation in critically ill patients: Systematic review. Med. Intensiv..

[23] Trethewey S.P., Brown N., Gao F., Turner A.M. (2019). Interventions for the management and prevention of sarcopenia in the critically ill: A systematic review. J. Crit. Care.

[24] Dobšák P., Tomandl J., Spinarova L., Vitovec J., Dusek L., Novakova M., Jarkovsky J., Krejci J., Hude P., Honek T. (2012). Effects of Neuromuscular Electrical Stimulation and Aerobic Exercise Training on Arterial Stiffness and Autonomic Functions in Patients with Chronic Heart Failure. Artif. Organs..

[25] Peters M.D.J., Marnie C., Tricco A.C., Pollock D., Munn Z., Alexander L., McInerney P., Godfrey C.M., Khalil H. (2020). Updated methodological guidance for the conduct of scoping reviews. JBI Evid. Synth..

[26] Tricco A.C., Lillie E., Zarin W., O’Brien K.K., Colquhoun H., Levac D., Moher D., Peters M.D.J., Horsley T., Weeks L. (2018). PRISMA Extension for Scoping Reviews (PRISMA-ScR): Checklist and Explanation. Ann. Intern. Med..

[27] Haddaway N.R., Grainger M.J., Gray C.T. (2022). Citationchaser: A tool for transparent and efficient forward and backward citation chasing in systematic searching. Res. Synth. Methods.

[28] Ouzzani M., Hammady H., Fedorowicz Z., Elmagarmid A. (2016). Rayyan—A web and mobile app for systematic reviews. Syst. Rev..

[29] Vanhorebeek I., Gunst J., Casaer M.P., Derese I., Derde S., Pauwels L., Segers J., Hermans G., Gosselink R., Berghe G.V.D. (2023). Skeletal Muscle Myokine Expression in Critical Illness, Association with Outcome and Impact of Therapeutic Interventions. J. Endocr. Soc..

[30] Grunow J.J., Reiher K., Carbon N.M., Engelhardt L.J., Mai K., Koch S., Schefold J.C., Z’Graggen W., Schaller S.J., Fielitz J. (2022). Muscular myostatin gene expression and plasma concentrations are decreased in critically ill patients. Crit. Care..

[31] Wollersheim T., Grunow J.J., Carbon N.M., Haas K., Malleike J., Ramme S.F., Schneider J., Spies C.D., Märdian S., Mai K. (2019). Muscle wasting and function after muscle activation and early protocol-based physiotherapy: An explorative trial. J. Cachexia Sarcopenia Muscle.

[32] Winkelman C., Sattar A., Momotaz H., Johnson K.D., Morris P., Rowbottom J.R., Thornton J.D., Feeney S., Levine A. (2018). Dose of Early Therapeutic Mobility: Does Frequency or Intensity Matter?. Biol. Res. Nurs..

[33] Winkelman C., Higgins P.A., Chen Y.J.K., Levine A.D. (2007). Cytokines in chronically critically ill patients after activity and rest. Biol. Res. Nurs..

[34] Winkelman C., Johnson K.D., Gordon N. (2015). Associations Between Muscle-Related Cytokines and Selected Patient Outcomes in the ICU. Biol. Res. Nurs..

[35] Winkelman C., Johnson K.D., Hejal R., Gordon N.H., Rowbottom J., Daly J., Peereboom K., Levine A.D. (2012). Examining the positive effects of exercise in intubated adults in ICU: A prospective repeated measures clinical study. Intensive Crit. Care Nurs..

[36] Silva P.E., Marqueti R.d.C., Livino-De-Carvalho K., de Araujo A.E.T., Castro J., da Silva V.M., Vieira L., Souza V.C., Dantas L.O., Cipriano G. (2019). Neuromuscular electrical stimulation in critically ill traumatic brain injury patients attenuates muscle atrophy, neurophysiological disorders, and weakness: A randomized controlled trial. J. Intensive Care.

[37] Kayambu G., Boots R., Paratz J. (2015). Early physical rehabilitation in intensive care patients with sepsis syndromes: A pilot randomised controlled trial. Intensive Care Med..

[38] Jonkman A.H., Frenzel T., McCaughey E.J., McLachlan A.J., Boswell-Ruys C.L., Collins D.W., Gandevia S.C., Girbes A.R.J., Hoiting O., Kox M. (2020). Breath-synchronized electrical stimulation of the expiratory muscles in mechanically ventilated patients: A randomized controlled feasibility study and pooled analysis. Crit. Care..

[39] França E., Gomes J., De Lira J., Amaral T., Vilaça A., Júnior M.P., Júnior U.E., Júnior L.F., Costa M., Andrade M. (2020). Acute effect of passive cycle-ergometry and functional electrical stimulation on nitrosative stress and inflammatory cytokines in mechanically ventilated critically ill patients: A randomized controlled trial. Braz. J. Med. Biol. Res..

[40] de França E.E.T., Ribeiro L.C., Lamenha G.G., Magalhães I.K.F., Figueiredo T.d.G., Costa M.J.C., Júnior U.F.E., Feitosa B.L., Andrade M.D.A., Júnior M.A.V.C. (2017). Oxidative stress and immune system analysis after cycle ergometer use in critical patients. Clinics.

[41] Files D., Morris P., Shrestha S., Dhar S., Young M., Hauser J., Chmelo E., Thompson C., Dixon L., Murphy K. (2013). Randomized, controlled pilot study of early rehabilitation strategies in acute respiratory failure. Crit. Care..

[42] Carvalho M.T.X., Real A.A., Cabeleira M.E., Schiling E., Lopes I., Bianchin J., da Silva A.M.V., Annoni R., de Albuquerque I.M. (2020). Acute effect of passive cycling exercise on serum levels of interleukin-8 and interleukin-10 in mechanically ventilated critically ill patients. Int. J. Ther. Rehabil..

[43] Bloch S., Syburrah T., Rosendahl U., Kemp P., Griffiths M., Polkey M. (2014). A paradoxical rise in rectus femoris myostatin (GDF-8) and GDF-15 in response to neuromuscular electrical stimulation in critical care. Thorax.

[44] Amidei C., Sole M.L. (2013). Physiological Responses to Passive Exercise in Adults Receiving Mechanical Ventilation. Am. J. Crit. Care.

[45] Akar O., Günay E., Ulasli S.S., Ulasli A.M., Kacar E., Sariaydin M., Solak Ö., Celik S., Ünlü M. (2017). Efficacy of neuromuscular electrical stimulation in patients with COPD followed in intensive care unit. Clin. Respir. J..

[46] Pekkala S., Wiklund P.K., Hulmi J.J., Ahtiainen J.P., Horttanainen M., Pöllänen E., Mäkelä K.A., Kainulainen H., Häkkinen K., Nyman K. (2013). Are skeletal muscle FNDC5 gene expression and irisin release regulated by exercise and related to health?. J. Physiol..

[47] Reza M.M., Subramaniyam N., Sim C.M., Ge X., Sathiakumar D., McFarlane C., Sharma M., Kambadur R. (2017). Irisin is a pro-myogenic factor that induces skeletal muscle hypertrophy and rescues denervation-induced atrophy. Nat. Commun..

[48] Schlittler M., Goiny M., Agudelo L.Z., Venckunas T., Brazaitis M., Skurvydas A., Kamandulis S., Ruas J.L., Erhardt S., Westerblad H. (2016). Endurance exercise increases skeletal muscle kynurenine aminotransferases and plasma kynurenic acid in humans. Am. J. Physiol. Cell Physiol..

[49] Kleinert M., Clemmensen C., Sjøberg K.A., Carl C.S., Jeppesen J.F., Wojtaszewski J.F., Kiens B., Richter E.A. (2018). Exercise increases circulating GDF15 in humans. Mol. Metab..

[50] Adela R., Banerjee S.K. (2015). GDF-15 as a target and biomarker for diabetes and cardiovascular diseases: A translational prospective. J. Diabetes Res..

[51] Kanzleiter T., Rath M., Görgens S.W., Jensen J., Tangen D.S., Kolnes A.J., Kolnes K.J., Lee S., Eckel J., Schürmann A. (2014). The myokine decorin is regulated by contraction and involved in muscle hypertrophy. Biochem. Biophys. Res. Commun..

