# Supplementary material for: Myokine Secretion Dynamics and Their Role in Critically Ill Patients: A Scoping Review
Source: J Clin Med. 2025 Apr 23;14(9):2892. doi: 10.3390/jcm14092892 (PMC12072662; doi:10.3390/jcm14092892)
Supplement: Supplementary file 1 [file jcm-14-02892-s001.zip › jcm-3429562-supplementary.pdf]

SUPPLEMENTARY MATERIAL

Table S1. Search strategy.

Database	N°	Search term
MEDLINE, using the Ovid platform	1	exp Cytokines/
	2	exp Chemokines/
	3	exp Interleukins/
	4	exp Biomarkers/
	5	(cytokin$ or chemokin$ or interleukin$ or miokin$ or exerkin$ or biomarker$ or biologic$ marker$).mp,tw.
	6	(Angiopoietin-like 4 or ANGPTL4 or Apelin or aminoisobutyric acid or BAIBA or Brain-derived neurotrophic factor or BDNF or Chemokine ligand or C-X-C motif or Decorin or Fibroblast growth factor 21 or FGF21 or IL-6 or IL-8 or IL-10 or IL-13 or IL-15 or IL-18 or Irisin or FNDC5 or Musclin or Myonectin or C1QTNF5 or Myostatin or Leukemia inhibitory factor or LIF or Secreted protein acidic rich in cysteine or SPARC or Tumor necrosis factor$ or TNF$).mp,tw.
	7	or/1-6
	8	exp intensive Care/
	9	exp critical Illness/
	10	exp Critical Care/
	11	exp Intensive Care Units/
	12	((intensive or critic$) adj2 (care or ill$)).mp,tw.
	13	exp Respiration, Artificial/
	14	exp Ventilator Weaning/
	15	((mechanic$ or artificial) adj2 (ventilat$ or respirat$)).mp,tw.
	16	or/8-15
	17	exp Electric Stimulation Therapy/
	18	exp Transcutaneous Electric Nerve Stimulation/
	19	exp Electric Stimulation/
	20	((neuromusc$ or functional or transcutan$) adj2 (electric$ or electrotherap$)).mp,tw.
	21	(electrotherap$ or electromyostimulation or electrostimulation or (electric$ adj2 stimulation)).mp,tw.
	22	(NMES or FES or TENS).mp,tw.
	23	or/17-22
	24	exp Exercise/
	25	exp Exercise Therapy/
	26	exp Physical Therapy Modalities/
	27	exp Occupational Therapy/
	28	exp Muscle Contraction/
	29	exp Resistance Training/
	30	(physiotherap$ or physical therap$ or kinesiotherap$ or physical rehabilitation or exercis$ or train$).mp,tw.
	31	(musc$ adj2 contrac$).mp,tw.
	32	or/24-31

	33	or/23,32
	34	and/7,16,33
Embase, using the Ovid platform	1	exp Cytokine/
	2	exp Chemokine/
	3	exp interleukin derivative/
	4	exp biological marker/
	5	(cytokin$ or chemokin$ or interleukin$ or miokin$ or exerkin$ or biomarker$ or biologi$ marker$).mp,tw.
	6	(Angiopoietin-like 4 or ANGPTL4 or Apelin or aminoisobutyric acid or BAIBA or Brain-derived neurotrophic factor or BDNF or Chemokine ligand or C-X-C motif or Decorin or Fibroblast growth factor 21 or FGF21 or IL-6 or IL-8 or IL-10 or IL-13 or IL-15 or IL-18 or Irisin or FNDC5 or Musclin or Myonectin or C1QTNF5 or Myostatin or Leukemia inhibitory factor or LIF or Secreted protein acidic rich in cysteine or SPARC or Tumor necrosis factor$ or TNF$).mp,tw.
	7	or/1-6
	8	exp intensive Care/
	9	exp critical Illness/
	10	exp Intensive Care Unit/
	11	((intensive or critic$) adj2 (care or ill$)).mp,tw.
	12	exp artificial ventilation/
	13	exp Ventilator Weaning/
	14	((mechanic$ or artificial) adj2 (ventilat$ or respirat$)).mp,tw.
	15	or/8-14
	16	exp electrotherapy/
	17	exp Transcutaneous Electric Nerve Stimulation/
	18	exp electrostimulation/
	19	((neuromusc$ or functional or transcutan$) adj2 (electric$ or electrotherap$)).mp,tw.
	20	(electrotherap$ or electromyostimulation or electrostimulation or (electric$ adj2 stimulation)).mp,tw.
	21	(NMES or FES or TENS).mp,tw.
	22	or/16-21
	23	exp Exercise/
	24	exp kinesiotherapy/
	25	exp physiotherapy/
	26	exp Occupational Therapy/
	27	exp Muscle Contraction/
	28	exp Resistance Training/
	29	(physiotherap$ or physical therap$ or kinesiotherap$ or physical rehabilitation or exercis$ or train$).mp,tw.
	30	(musc$ adj2 contrac$).mp,tw.
	31	or/23-30
	32	or/22,31
	33	and/7,15,32
	1	(MH "Cytokines+")

CINAHL, using the EBSCOhost platform	2	(MH "Chemokines+")
	3	(MH "Interleukins+")
	4	(MH "Biological Markers+")
	5	cytokin* or chemokin* or interleukin* or miokin* or exerkin* or biomarker* or biologic* marker*
	6	Angiopoietin-like 4 or ANGPTL4 or Apelin or aminoisobutyric acid or BAIBA or Brain-derived neurotrophic factor or BDNF or Chemokine ligand or C-X-C motif or Decorin or Fibroblast growth factor 21 or FGF21 or IL-6 or IL-8 or IL-10 or IL-13 or IL-15 or IL-18 or Irisin or FNDC5 or Musclin or Myonectin or C1QTNF5 or Myostatin or Leukemia inhibitory factor or LIF or Secreted protein acidic rich in cysteine or SPARC or Tumor necrosis factor* or TNF*
	7	S1 OR S2 OR S3 OR S4 OR S5 OR S6
	8	(MH "Critical Care+")
	9	(MH "Critical Illness")
	10	(MH "Intensive Care Units+")
	11	(intensive or critic*) N2 (care or ill*)
	12	(MH "Respiration, Artificial+")
	13	(MH "Ventilator Weaning")
	14	(mechanic* or artificial) N2 (ventilat* or respirat*)
	15	S8 OR S9 OR S10 OR S11 OR S12 OR S13 OR S14
	16	(MH "Electric Stimulation+")
	17	(MH "Transcutaneous Electric Nerve Stimulation")
	18	(MH "Electrical Stimulation, Neuromuscular")
	19	(MH "Electrical Stimulation, Functional")
	20	(neuromusc* or functional or transcutan*) N2 (electric* or electrotherap*)
	21	electrotherap* or electromyostimulation or electrostimulation or (electric* N2 stimulation)
	22	NMES or FES or TENS
	23	S16 OR S17 OR S18 OR S19 OR S20 OR S21 OR S22
	24	(MH "Exercise+")
	25	(MH "Therapeutic Exercise+")
	26	(MH "Physical Therapy+")
	27	(MH "Occupational Therapy+")
	28	(MH "Muscle Contraction+")
	29	(MH "Resistance Training")
	30	physiotherap* or physical therap* or kinesiotherap* or physical rehabilitation or exercis* or train*
	31	musc* N2 contrac*
	32	S24 OR S25 OR S26 OR S27 OR S28 OR S29 OR S30 OR S31
	33	S23 OR S32
	34	S7 AND S15 AND S33
Scopus		(TITLE-ABS-KEY (cytokin* OR chemokin* OR interleukin* OR miokin* OR exerkin* OR biomarker* OR "biologic* marker*" OR "angioived neurotropoietin-like 4" OR angptl4 OR apelin OR "aminoisobutyric acid" OR baiba OR "brain-derphic factor" OR bdnf OR "chemokine ligand" OR "c-x-c motif" OR decorin OR "fibroblast growth factor 21"

	OR fgf21 OR il-6 OR il-8 OR il-10 OR il-13 OR il-15 OR il-18 OR irisin OR fndc5 OR musclin OR myonectin OR c1qtnf5 OR myostatin OR "leukemia inhibitory factor" OR lif OR "secreted protein acidic rich in cysteine" OR sparc OR "tumor necrosis factor*" OR tnf*) AND TITLE-ABS-KEY (((intensive OR critic*) W/2 (care OR ill*)) OR ((mechanic* OR artificial) W/2 (ventilat* OR respirat*))) AND TITLE-ABS-KEY (((neuromusc* OR functional OR transcutan*) W/2 (electric* OR electrotherap*)) OR (electrotherap* OR electromyostimulation OR electrostimulation OR (electric* W/2 stimulation)) OR (nmes OR fes OR tens) OR (physiotherap* OR "physical therap*" OR kinesiotherap* OR "physical rehabilitation" OR exercis* OR train*) OR (musc* W/2 contrac*)))
WoS	cytokin* OR chemokin* OR interleukin* OR miokin* OR exerkin* OR biomarker* OR "biologic* marker*" OR "angioived neurotropoietin-like 4" OR angptl4 OR apelin OR "aminoisobutyric acid" OR barba OR "brain-derphic factor" OR bdnf OR "chemokine ligand" OR "c-x-c motif" OR decorin OR "fibroblast growth factor 21" OR fgf21 OR il-6 OR il-8 OR il-10 OR il-13 OR il-15 OR il-18 OR irisin OR fndc5 OR muscling OR mionectine OR c1qtnf6 OR myostatin OR "leukemia inhibitory factor" OR lif OR "secreted protein acidic rich in cysteine" OR sparc OR "tumor necrosis factor*" OR tnf* (Topic) and ((intensive OR critic*) NEAR/2 (care OR ill*)) OR ((mechanic* OR artificial) NEAR/2 (ventilat* OR respirat*)) (Topic) and ((neuromusc* OR functional OR transcutan*) NEAR/2 (electric* OR electrotherap*)) OR (electrotherap* OR electromyostimulation OR electrostimulation OR (electric* NEAR/2 stimulation)) OR (nmes OR fes OR tens) OR (physiotherap* OR "physical therap*" OR kinesiotherap* OR "physical rehabilitation" OR exercis* OR train*) OR (musc* NEAR/2 contrac*) (Topic)

Table S2. Reasons for exclusion of full-text evaluated studies.

ID	Title	DOI	Reason
Ahn 2018	Effects of Early Exercise Rehabilitation on Functional Recovery in Patients with Severe Sepsis	10.3349/ymj.2018.59.7.843	Concept: Concentration of myokines related to muscle contraction is not assessed
Amidei 2012	Measurement of physiologic responses to mobilisation in critically ill adults	10.1016/j.iccn.2011.09.002	Study design: Narrative review
Armbruster 2010	In vitro muscle contraction force measurements on isolated and entire rat diaphragms		Population: Animal model study
Arcanjo 2021	Critically ill COVID-19 patients exhibit hyperactive cytokine responses associated with effector exhausted senescent T cells in acute infection	10.1093/infdis/jiab425	Concept: Concentration of myokines related to muscle contraction is not assessed
Azcona 2014	Randomized trial of non-invasive ventilation combined with exercise training in patients with chronic hypercapnic failure due to chronic obstructive pulmonary disease	10.1016/j.rmed.2014.10.005	Population: Non-critically ill patients
Azevedo 2022	Muscle weakness in critically ill patients: Effects of a systematized rehabilitation nursing program	10.1016/j.enfcle.2022.11.001	Concept: Concentration of myokines related to muscle contraction is not assessed
Baldwin 2014	Alterations in respiratory and limb muscle strength and size in patients with sepsis who are mechanically ventilated	10.2522/ptj.20130048	Concept: Concentration of myokines related to muscle contraction is not assessed
Bao 2022	Prevention of muscle atrophy in ICU patients without nerve injury by neuromuscular electrical stimulation: a randomized controlled study	10.1186/s12891-022-05739-2	Concept: Concentration of myokines related to

			muscle contraction is not assessed
Baron 2022	Efficacy and safety of neuromuscular electrical stimulation in the prevention of pressure injuries in critically ill patients: a randomized controlled trial	10.1186/s13613-022-01029-1	Concept: Concentration of myokines related to muscle contraction is not assessed
Barnett 2018	Re: Aerobic Exercise Combined with Noninvasive Positive Pressure Ventilation Increases Serum Brain-Derived Neurotrophic Factor in Healthy Males by Kawazu et al.	10.1016/j.pmrj.2018.03.011	Study design: Letter to the editor
Berney 2021	Functional electrical stimulation in-bed cycle ergometry in mechanically ventilated patients: a multicentre randomised controlled trial	10.1136/thoraxjnl-2020-215093	Concept: Concentration of myokines related to muscle contraction is not assessed
Brainin 2003	Physical exercise and stroke: The sitting majority has a lesson to learn	10.1161/01.STR.0000091843.02517.9D	Study design: Systematic review
Campos 2022	Early Neuromuscular Electrical Stimulation in Addition to Early Mobilization Improves Functional Status and Decreases Hospitalization Days of Critically Ill Patients	10.1097/ccm.0000000000005557	Concept: Concentration of myokines related to muscle contraction is not assessed
Carilho 2013	Vascular endothelial growth factor and amyotrophic lateral sclerosis: The interplay with exercise and noninvasive ventilation	10.1002/mus.23955	Population: Non-critically ill patients
Carvalho 2022	Multidisciplinary rehabilitation in intensive care for COVID-19: randomised controlled trial	10.1183/23120541.00350-2022	Concept: Concentration of myokines related to muscle contraction is not assessed
De Letter 2000	Critical illness polyneuropathy and myopathy (CIPNM): evidence for local immune activation by cytokine-expression in the muscle tissue	10.1016/s0165-5728(99)00252-0	Concept: Concentration of myokines related to muscle contraction is not assessed

Delfin 2013	Critical illness polyneuromyopathy presenting as bulbar palsy: A case report	10.1378/chest.1704196	Concept: Concentration of myokines related to muscle contraction is not assessed
Denehy 2013	Exercise rehabilitation for patients with critical illness: a randomized controlled trial with 12 months of follow-up	10.1186/cc12835	Concept: Concentration of myokines related to muscle contraction is not assessed
Eggmann 2018	Effects of early, combined endurance and resistance training in mechanically ventilated, critically ill patients: A randomised controlled trial	10.1371/journal.pone.0207428	Concept: Concentration of myokines related to muscle contraction is not assessed
Elliott 2016	Is rehabilitation post critical illness a new anti-inflammatory agent?	10.1136/thoraxjnl-2016-208844	Study design: Editorial
Ferraro 2021	COVID-19 related fatigue: Which role for rehabilitation in post-COVID-19 patients? A case series	10.1002/jmv.26717	Concept: Concentration of myokines related to muscle contraction is not assessed
Ferreira 2013	Effects of electrical muscle stimulation early in the quadriceps and tibialis anterior muscle of critically ill patients	10.3109/09593985.2013.869773	Concept: Concentration of myokines related to muscle contraction is not assessed
Fischer 2016	Muscle mass, strength and functional outcomes in critically ill patients after cardiothoracic surgery: does neuromuscular electrical stimulation help? The Catastim 2 randomized controlled trial	10.1186/s13054-016-1199-3	Concept: Concentration of myokines related to muscle contraction is not assessed
Fossat 2018	Effect of In-Bed Leg Cycling and Electrical Stimulation of the Quadriceps on Global Muscle Strength in Critically Ill Adults: A Randomized Clinical Trial	10.1001/jama.2018.9592	Concept: Concentration of myokines related to muscle contraction is not assessed

Gao 2018	Transcutaneous electrical acupoint stimulation for prevention of postoperative delirium in geriatric patients with silent lacunar infarction: A preliminary study	10.2147/CIA.S183698	Concept: Concentration of myokines related to muscle contraction is not assessed
Gerovasili 2009	Short-term Systemic Effect of Electrical Muscle Stimulation in Critically Ill Patients	10.1378/chest.08-2888	Concept: Concentration of myokines related to muscle contraction is not assessed
Giallauria 2006	Reduction of N terminal-pro-brain (B-type) natriuretic peptide levels with exercise-based cardiac rehabilitation in patients with left ventricular dysfunction after myocardial infarction		Population: Non-critically ill patients
Grieco 2022	Individualized positive end-expiratory pressure guided by end-expiratory lung volume in early acute respiratory distress syndrome: study protocol for the multicenter, randomized IPERPEEP trial.	10.1186/s13063-021-05993-0	Study design: Study protocol
Griffin 2009	Functional electrical stimulation cycling improves body composition, metabolic and neural factors in persons with spinal cord injury	10.1016/j.jelekin.2008.03.002	Population: Non-critically ill patients
Gruther 2010	Effects of neuromuscular electrical stimulation on muscle layer thickness of knee extensor muscles in intensive care unit patients: a pilot study	10.2340/16501977-0564	Concept: Concentration of myokines related to muscle contraction is not assessed
Gutierrez 2022	Critically Ill Patients Treated for Chimeric Antigen Receptor-Related Toxicity: A Multicenter Study	10.1097/CCM.0000000000005149	Concept: Concentration of myokines related to muscle contraction is not assessed
Hannink 2014	Non-invasive ventilation abolishes the IL-6 response to exercise in muscle-wasted COPD patients: A pilot study	10.1111/j.1600-0838.2012.01484.x	Population: Non-critically ill patients

Ioannis 2019	Effect of Different Neuromuscular Electrical Stimulation Protocols on Muscle Mass in Intensive Care Unit Patients: A Pilot Study		Concept: Concentration of myokines related to muscle contraction is not assessed
Jian-biao 2018	Electroacupuncture Improves Intestinal Dysfunction in Septic Patients: A Randomised Controlled Trial	10.1155/2018/8293594	Concept: Concentration of myokines related to muscle contraction is not assessed
Kagan 2022	Effect of Combined Protein-Enriched Enteral Nutrition and Early Cycle Ergometry in Mechanically Ventilated Critically Ill Patients-A Pilot Study	10.3390/nu14081589	Concept: Concentration of myokines related to muscle contraction is not assessed
Kawazu 2016	Aerobic Exercise Combined with Noninvasive Positive Pressure Ventilation Increases Serum Brain-Derived Neurotrophic Factor in Healthy Males	10.1016/j.pmrj.2016.05.004	Population: Non-critically ill patients
Kho 2014	Neuromuscular electrical stimulation in mechanically ventilated patients: a randomized, sham-controlled pilot trial with blinded outcome assessment	10.1016/j.jcrc.2014.09.014	Concept: Concentration of myokines related to muscle contraction is not assessed
Kho 2016	TryCYCLE: A Prospective Study of the Safety and Feasibility of Early In-Bed Cycling in Mechanically Ventilated Patients	10.1371/journal.pone.0167561	Concept: Concentration of myokines related to muscle contraction is not assessed
Kim 2016	Differentiating Exercise-Induced Cardiac Adaptations From Cardiac Pathology: The "Grey Zone" of Clinical Uncertainty	10.1016/j.cjca.2015.11.025	Study design: Narrative review
Kohoutova 2019	Vagus Nerve Stimulation Attenuates Multiple Organ Dysfunction in Resuscitated Porcine Progressive Sepsis	10.1097/CCM.0000000000003714	Population: Animal model study
Machado 2017	Effects that passive cycling exercise have on muscle strength, duration of mechanical	10.1590/s1806-37562016000000170	Concept: Concentration of myokines related to

	ventilation, and length of hospital stay in critically ill patients: a randomized clinical trial		muscle contraction is not assessed
Maffei 2017	Intensive Early Rehabilitation in the Intensive Care Unit for Liver Transplant Recipients: A Randomized Controlled Trial	10.1016/j.apmr.2017.01.028	Concept: Concentration of myokines related to muscle contraction is not assessed
Marquez-Martin 2012	Home mechanical ventilation and respiratory rehabilitation: Influence in bode index, quality of live and inflammatory parameters	10.1164/ajrccm-conference.2012.185.1_MeetingAbstracts.A5786	Population: Non-critically ill patients
Medrinal 2018	Comparison of exercise intensity during four early rehabilitation techniques in sedated and ventilated patients in ICU: a randomised cross-over trial	10.1186/s13054-018-2030-0	Concept: Concentration of myokines related to muscle contraction is not assessed
Mohan 2012	Effect of exercise on the vital signs and inflammatory biomarkers: Scientific views	10.1016/j.iccn.2012.02.007	Study design: Letter to the editor
Nakamura 2019	Efficacy of belt electrode skeletal muscle electrical stimulation on reducing the rate of muscle volume loss in critically ill patients: A randomized controlled trial	10.2340/16501977-2594	Concept: Concentration of myokines related to muscle contraction is not assessed
Nakanishi 2020	Effect of Electrical Muscle Stimulation on Upper and Lower Limb Muscles in Critically Ill Patients: A Two-Center Randomized Controlled Trial	10.1097/ccm.0000000000004522	Concept: Concentration of myokines related to muscle contraction is not assessed
Nakano 2022	Novel protocol combining physical and nutrition therapies, Intensive Goal-directed REhabilitation with Electrical muscle stimulation and Nutrition (IGREEN) care bundle	10.1186/s13054-021-03827-8	Concept: Concentration of myokines related to muscle contraction is not assessed
Nickels 2020	Effect of in-bed cycling on acute muscle wasting in critically ill adults: A randomised clinical trial	10.1016/j.jcrc.2020.05.008	Concept: Concentration of myokines related to muscle contraction is not assessed

Nydahl 2019	PROtocol-based MObilizaTION on intensive care units: stepped-wedge, cluster-randomized pilot study (Pro-Motion)	10.1111/nicc.12438	Concept: Concentration of myokines related to muscle contraction is not assessed
Nydahl 2021	Mobilization in the evening to prevent delirium: A pilot randomized trial	10.1111/nicc.12638	Concept: Concentration of myokines related to muscle contraction is not assessed
Nozoe 2018	Neuromuscular electrical stimulation is ineffective for treating quadriceps muscle wasting with ruptured aneurysm: A case report	10.1016/j.amsu.2018.09.011	Concept: Concentration of myokines related to muscle contraction is not assessed
Olof 2019	Effects of intensive upright mobilisation on outcomes of mechanically ventilated patients in the intensive care unit: a randomised controlled trial with 12-months follow-up	10.1080/21679169.2019.1645880	Concept: Concentration of myokines related to muscle contraction is not assessed
Poulsen 2011	Effect of transcutaneous electrical muscle stimulation on muscle volume in patients with septic shock	10.1097/ccm.0b013e318205c7bc	Concept: Concentration of myokines related to muscle contraction is not assessed
Powers 2013	CrossTalk proposal: Mechanical ventilation-induced diaphragm atrophy is primarily due to inactivity	10.1113/jphysiol.2013.254680	Study design: Report of an expert discussion
Ravan 2017	Investigating the correlation between short-term effectiveness of VNS Therapy in reducing the severity of seizures and long-term responsiveness	10.1016/j.eplepsyres.2017.04.008	Population: Non-critically ill patients
Rezaeikia 2020	Effect of Passive Movements of Lower Extremity on Hemodynamic Parameters of the Patients under Ventilator	10.52547/jccs.1.1.37	Concept: Concentration of myokines related to muscle contraction is not assessed

Routsi 2010	Electrical muscle stimulation prevents critical illness polyneuromyopathy: a randomized parallel intervention trial	10.1186/cc8987	Concept: Concentration of myokines related to muscle contraction is not assessed
Samadi 2020	A study of possible role of exercise and some antioxidant supplements against coronavirus disease 2019 (COVID-19): A cytokines related perspective	10.1016/j.apunsm.2020.06.003	Study design: Letter to the editor
Sanchis-Gomar 2019	Neuromuscular Electrical Stimulation: A New Therapeutic Option for Chronic Diseases Based on Contraction-Induced Myokine Secretion	10.3389/fphys.2019.01463	Study design: Narrative review
Santos 2019	Acute effect of passive cycloergometry on the cardiovascular system and respiratory mechanics of critically ill patients: a randomized controlled trial	10.1590/1980-5918.032.ao32	Concept: Concentration of myokines related to muscle contraction is not assessed
Segers 2020	Early neuromuscular electrical stimulation reduces the loss of muscle mass in critically ill patients - A within subject randomized controlled trial	10.1016/j.jcrc.2020.11.018	Concept: Concentration of myokines related to muscle contraction is not assessed
Sellareés 2012	The effect of spontaneous breathing on systemic interleukin-6 during ventilator weaning	10.1183/09031936.00037511	Concept: Concentration of myokines related to muscle contraction is not assessed
Seo 2019	Feasibility, safety, and functional recovery after active rehabilitation in critically ill surgical patients	10.1016/j.aucc.2019.07.005	Concept: Concentration of myokines related to muscle contraction is not assessed
Silva 2016	Safety and feasibility of a neuromuscular electrical stimulation chronaxie-based protocol in critical ill patients: A prospective observational study	10.1016/j.jcrc.2016.09.012	Concept: Concentration of myokines related to muscle contraction is not assessed

Strommen 2013	Intensive treadmill training and cytokine response in acute ischemic stroke		Population: Non-critically ill patients
Tamvaki 2018	Pain Neuropeptides Plasma Levels' Measurement as Biomarkers of Opioids Effectiveness in Critically ill Children		Population: Paediatric patients
van Blydenstein 2021	Prevalence and Trajectory of COVID-19-Associated Hypercoagulability Using Serial Thromboelastography in a South African Population	10.1155/2021/3935098	Concept: Concentration of myokines related to muscle contraction is not assessed
Waldauf 2021	Functional electrical stimulation-assisted cycle ergometry-based progressive mobility programme for mechanically ventilated patients: randomised controlled trial with 6 months follow-up	10.1136/thoraxjnl-2020-215755	Concept: Concentration of myokines related to muscle contraction is not assessed
Wang 2018	Improving Recovery and Outcomes Every Day after the ICU (IMPROVE): study protocol for a randomized controlled trial	10.1186/s13063-018-2569-8	Study design: Study protocol
Winkelman 2007	Inactivity and inflammation in the critically ill patient	10.1016/j.ccc.2006.11.002	Study design: Narrative review
Xinli 2012	Cardioprotective effect of transcutaneous electric acupoint stimulation in the pediatric cardiac patients: A randomized controlled clinical trial	10.1111/j.1460-9592.2012.03822.x	Population: Paediatric patients
Yamakawa 2013	Electrical Vagus Nerve Stimulation Attenuates Systemic Inflammation and Improves Survival in a Rat Heatstroke Model	10.1371/journal.pone.0056728	Population: Animal model study
Yousef 2014	The Value of Admission Serum IL-8 Monitoring and the Correlation with IL-8 (-251A/T) Polymorphism in Critically Ill Patients	10.1155/2014/494985	Concept: Concentration of myokines related to muscle contraction is not assessed
Yosef-Brauner 2014	Effect of physical therapy on muscle strength, respiratory muscles and functional parameters in	10.1111/crj.12091	Concept: Concentration of myokines related to

	patients with intensive care unit-acquired weakness		muscle contraction is not assessed
Zhao 2020	Effect of Transcutaneous Electrical Acupoint Stimulation on One-Lung Ventilation-Induced Lung Injury in Patients Undergoing Esophageal Cancer Operation	10.1155/2020/9018701	Population: These are cancer patients studied during oesophageal cancer surgery
Zong-xiu 2022	A combined risk model for the multi-encompassing identification of heterogeneities of prognoses, biological pathway variations and immune states for sepsis patients	10.1186/s12871-021-01552-x	Concept: Concentration of myokines related to muscle contraction is not assessed
